# Total Contact Cast after Sole Free Flap Reconstruction for Early Ambulation

**DOI:** 10.1055/s-0044-1800813

**Published:** 2025-03-11

**Authors:** Yutaro Yamashita, Yoshiro Abe, Mayu Bando, Shunsuke Mima, Hiroyuki Yamasaki, Shinji Nagasaka, Kazuhide Mineda, Ichiro Hashimoto

**Affiliations:** 1 Department of Plastic Reconstructive and Aesthetic Surgery, The University of Tokushima, Tokushima, Japan

**Keywords:** chronic limb-threatening ischemia, total contact cast, free flap, diabetic foot ulcer

## Abstract

**Background** Free flap reconstruction can be performed in patients with chronic limb-threatening ischemia (CLTI). However, early walking training may increase the risk of wound dehiscence and prolong hospitalization. Total contact cast (TCC) treatment effectively addresses diabetic plantar ulcers by immobilizing the foot and distributing weight away from the ulcer area. This study aimed to assess the effect of postoperative TCC use on early limb loading and hospital stay in patients with CLTI with free flaps.

**Methods** Patients with CLTI who underwent free flap reconstruction between 2006 and 2023 were enrolled in this study. Postoperative time until weight-bearing initiation was compared between the TCC ( *n* = 5) and non-TCC groups ( *n* = 7).

**Results** The time to the initiation of weight-bearing on the affected limb was 52.3 ± 33.2 days in the non-TCC group and 19.8 ± 3.56 days in the TCC group ( *p* = 0.105). The wound dissection rates were 42.9% (3/7) in the non-TCC group and 20% (1/5) in the TCC group ( *p* = 0.408). At discharge, 28.6% (2/7) of the non-TCC group and 20% (1/5) of the TCC group had ulcers ( *p* = 0.735). The average flap size was 149 ± 69.1 cm ^2^ in the non-TCC group and 95.6 ± 73.1 cm ^2^ in the TCC group ( *p* = 0.268).

**Conclusion** Postoperative TCC use after free flap foot reconstruction may lead to early weight-bearing of the affected limb. Further studies with larger numbers of cases are needed.

## Introduction

With the increasing prevalence of diabetes worldwide, the number of patients with chronic limb-threatening ischemia (CLTI) is increasing. 1 2 CLTI is considered to pose a risk of lower extremity amputation owing to ischemia, infection, and nerve damage. Its diagnosis requires that the patient has one of the following symptoms: (1) pain at rest confirmed by hemodynamic testing (Wound Ischemia Foot Infection [WIfI] grade 3 ischemia); (2) a diabetic ulcer or leg ulcer that lasts for more than 2 weeks; or (3) gangrene of the lower legs or feet. 3

Patients with CLTI are prone to foot ulcers and face a constant risk of lower limb amputation. 4 In cases where there is extensive bone exposure or weight-bearing areas, free flap reconstruction is performed. 5 6 7 8 9 10 As this is a reconstruction of the foot, determining the optimal time to resume walking can be challenging. Early walking training can lead to complications such as wound dehiscence and prolonged bed rest. When wound dehiscence occurs on the sole of the foot of a patient with CLTI, walking with full weight-bearing must be prohibited. Patients with CLTI have poor wound healing owing to diabetes, are prone to infection, and often require prolonged treatment. This means that the period of walking prohibition is long, and muscle weakness may occur. These complications can be addressed using a total contact cast (TCC), which is an effective treatment for diabetic plantar ulcers, immobilizing the foot and redistributing weight away from the ulcer area. 11 When weight is applied using a TCC, the pressure on the sole and heel can be significantly reduced. In addition, compared with other removable orthotics, a TCC is the most effective in the healing of diabetic foot ulcers. 12 For this reason, TCC is positioned as the gold standard treatment for diabetic foot ulcers. 13 14

The application of a TCC may enable early initiation of full weight-bearing in cases of CLTI reconstructed with free flaps, but the increase in complications associated with early weight-bearing and the use of a TCC is unknown. In this study, we aimed to investigate whether complications of the initiation of full weight-bearing were more common with the use of a TCC than those without and whether complications occurred owing to the fitting of a TCC.

## Methods

**Fig. 1:**
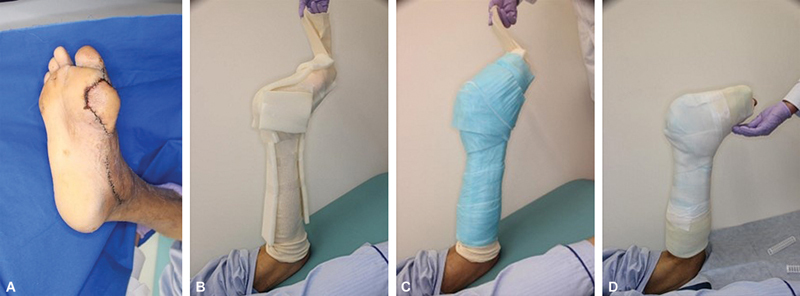
( **A** ) A case in which an anterolateral thigh flap was transplanted to the base of the big toe. ( **B** ) A cylindrical bandage was applied and the malleolus and midline were protected with a sponge. ( **C** ) Wrapping in a patting bandage. ( **D** ) Fixation using cast bandage.

We retrospectively investigated patients with CLTI and plantar ulcers who underwent free flap reconstruction between 2006 and 2023. This study was approved by the Ethics Committee of Tokushima University Hospital (approval number: 3943-2). Consent was obtained from the participants for the publication of their case details. We analyzed the postoperative complications and the number of days until weight-bearing in five cases with postoperative TCC (TCC group) and seven cases without TCC (non-TCC group). Since 2019, TCCs have been indicated for all cases of plantar ulcers reconstructed with free flaps in our institution. In the TCC group, by wearing the TCC, the load was distributed over the entire foot. Therefore, we thought that wound dissolution would be less likely to occur and we started loading earlier than that in the non-TCC group. Full weight-bearing was initiated approximately 2 weeks after surgery with the TCC applied. To reduce the risk of developing pressure ulcers due to the TCC, sponges were applied to bony protrusions such as the medial malleolus, lateral malleolus, heel, and tibia, and a cotton bandage was wrapped around the foot and lower leg. The ankle joint was fixed at approximately 90 degrees ( Fig. 1 ). In the TCC group, the timing of full weight loading was determined by the surgeon on the basis of the condition of the wound approximately 2 weeks later. The non-TCC group was determined on the basis of the condition of the wound approximately 4 weeks after surgery. In the non-TCC group, if no problems with the wound were detected, loading with a gauze dressing was started 4 weeks after surgery. In the TCC group, the TCC was removed after 1 week, and the skin flap was confirmed. Care was taken to avoid applying excessive pressure by covering the bypass vessels and the vascular anastomoses of the flap with a sponge. The color of the flap can be evaluated through the opening at the toe of the TCC in many cases. In cases of a heel flap, a hole can be made in the cast above the flap for such observation. Similarly, in the case of bypass vessels, a hole can be made in the desired area to evaluate the blood flow. The TCC was replaced once a week and applied continuously until approximately 1 month postoperatively. To closely examine osteomyelitis, an MRI examination was performed preoperatively. Contrast-enhanced computed tomography and ultrasound examinations were performed preoperatively in all cases. In addition, preoperative angiography was performed for cases in which revascularization was performed. We also compared flap size, hemodialysis, skin perfusion pressure (SPP), incidence of flap necrosis, incidence of wound dissection, and presence of ulcers at discharge between the two groups. Small ulcers that healed conservatively were not considered ulcers. We also investigated the presence of diabetes, WIfI stage, 15 Global Anatomic Staging System stage, 4 and the location of foot ulcers.

Statistical analysis was performed using Excel-based statistics, Mann–Whitney tests, and chi-square tests. Statistical significance was set at *p* < 0.05.

## Results

In the non-TCC group, the preoperative site of the foot ulcer was the plantar side in two cases, the forefoot in three cases, the heel in one case, and the lateral side of the foot in one case. In the TCC group, one ulcer involved the plantar side, three in the forefoot area, and one in the medial foot area. Transplanted flaps were present in the loading areas in all patients. All ulcers were stage 4 according to the WIfI classification ( Table 1 ), and all patients with CLTI had diabetes mellitus. The mean glycohemoglobin level was 7.58 ± 2.02% in the non-TCC group and 9.13 ± 3.39% in the TCC group ( *p* = 0.195). Four (57.1%) patients in the non-TCC group and two (40%) in the TCC group had hypertension. Additionally, three (42.9%) patients in the non-TCC group and two (40%) in the TCC group underwent hemodialysis ( *p* = 0.921). Four (57.1%) patients in the non-TCC group and four (80%) in the TCC group had a history of smoking. The average body mass index was 22.84 ± 7.32 kg/m ^2^ in the non-TCC group and 25.25 ± 1.58 kg/m ^2^ in the TCC group ( *p* = 0.315). The mean SPP was 50.6 ± 15.3 mm Hg in the TCC group and 67.8 ± 15.6 mm Hg in the non-TCC group ( *p* = 0.082). In the non-TCC group, foot ulcers occurred on the forefoot in two cases, midfoot in three cases, and heel in two cases. In the TCC group, foot ulcers occurred in the forefoot section of the foot in four cases, midfoot in one case, and heel in zero cases ( *p* = 0.175). In the non-TCC group, the Global Limb Anatomic Staging System stage was not applicable in one case, I in three cases, II in one case, and III in two cases ( *p* = 0.318; Table 2 ).

**Table 1:** Wound Ischemia Foot Infection grade

WIfI grade	Total *n* = 12	TCC group *n* = 5	non-TCC group *n* = 7
Wound 0 1 2 3	0 0 0 12	0 0 0 5	0 0 0 7
Ischemia 0 1 2 3	7 4 0 1	3 2 0 0	4 2 0 1
Foot infection 0 1 2 3	0 1 6 5	0 0 3 2	0 1 3 3

Abbreviations: TCC, total contact cast; WIfI, Wound Ischemia Foot Infection.

**Table 2:** Clinical characteristics of patients with chronic limb-threatening ischemia

Parameter	Total ( *n* = 12)	Non-TCC group ( *n* = 7)	TCC group ( *n* = 5)	*p* -value
Mean age ± SD, years(range)	58.3 ± 6.9 (49–69)	59.9 ± 6.9 (52–69)	56.2 ± 7.2 (49–67)	0.565
Sex, *N*				0.217
Men Women	11 1	7 0	4 1	
Diabetes, *N* (%) Mean HbA1c ± SD, %(range)	12 (100)	7 (100) 7.58 ± 2.02 (5.6–11.1)	5 (100) 9.13 ± 3.39 (5.7–13.1)	0.195
Hypertension, *N* (%)	6 (50)	4 (57.1)	2 (40)	0.558
Hemodialysis, *N* (%)	5 (41.7)	3 (42.9)	2 (40)	0.921
Smoking, *N* (%)	8 (66.7)	4 (57.1)	4 (80)	0.408
Mean body mass index ± SD, kg/m ^2^ (range)	23.72 ± 5.87 (15.9–36.2)	22.84 ± 7.32 (15.9–36.2)	25.25 ± 1.58 (23.2–27.0)	0.315
Skin perfusion pressure ± SD, mm Hg (range)	57.75 ± 17.18 (29–81)	50.57 ± 15.30 (29–75)	67.80 ± 15.64 (29–81)	0.082
Site of foot ulcer				0.175
Foremost part Middle foot Heel	6 4 2	2 3 2	4 1 0	
GLASS stage NA	4	1	3	0.318
I II III	4 2 2	3 1 2	1 1 0	

Abbreviations: GLASS, Global Limb Anatomic Staging System; HbA1c, glycohemoglobin; NA, not applicable; SD, standard deviation; TCC, total contact cast.

The average flap size was 149 ± 69.1 cm ^2^ in the non-TCC group and 95.6 ± 73.1 cm ^2^ in the TCC group ( *p* = 0.268). Regarding flaps, in the non-TCC group, six cases were reconstructed with the latissimus dorsi (LD) and one case with the anterolateral thigh (ALT). In the TCC group, the flaps used for reconstruction were the LD in two cases, ALT in two cases, and the scapula in one case. Regarding recipient vessels, the anterior tibial artery was selected in 11 cases, the posterior tibial artery in 1 case, and bypass grafting in 1 case ( Table 3 ). Partial flap necrosis was detected in three (42.9%) patients in the non-TCC group; however, it was not found in any patients in the TCC group ( *p* = 0.091). The postoperative wound dehiscence rate was 42.9% (3/7) and 20% (1/5) in the non-TCC and TCC groups, respectively ( *p* = 0.408). At the time of discharge, ulcers were detected in 28.6% (2/7) and 20% (1/5) of the patients in the non-TCC and TCC groups, respectively ( *p* = 0.735; Table 4 ). The postoperative time to the initiation of full weight-bearing on the affected limb was 52.3 ± 33.2 days in the non-TCC group and 19.8 ± 3.56 in the TCC group ( *p* = 0.105; Table 5 ). After discharge, the mean follow-up period was 78.1 months for the non-TCC group and 15 months for the TCC group. In the non-TCC group, four patients had ulcer recurrence in the follow-up period, and three underwent additional surgery. In the TCC group, one patient had a recurrence, and none of the patients underwent additional surgery.

**Table 3:** Surgical technique

Parameter	Total ( *n* = 12)	Non-TCC group ( *n* = 7)	TCC group ( *n* = 5)	*p* -value
Mean flap size ± SD, cm ^2^ (range)	126.8 ± 72.8 (28–270)	149 ± 69.1 (66–270)	95.6 ± 73.1 (28–216)	0.268
Type of flap				0.085
LD ALT Scapula	8 3 1	6 1	2 2 1	
Recipient vessel				0.206
ATA PTA Bypass graft	11 1 1	7 1 0	4 0 1	

Abbreviation: ALT, anterolateral thigh; ATA, anterior tibial artery; LD, latissimus dorsi; NA, not applicable; PTA, posterior tibial artery.

**Table 4:** Incidence of postoperative complications

Complications	Non-TCC group (%)	TCC group (%)	*p* -value
Flap partial necrosis	3/7 (42.9)	0/5 (0)	0.091
Wound dehiscence	3/7 (42.9)	1/5 (20%)	0.408
Ulcer remains at discharge	2/7 (28.6)	1/5 (20)	0.735

Abbreviation: TCC, total contact cast.

**Table 5:** Comparison of this study with previous reports

Study	Days until postoperative full weight-bearing (range)	Mean size of flap, cm ^2^ (range)	Site of ulcer	Postoperative complications	Pathology
TCC group (our study) *N* = 5	19.8 days (17–25)	95.6 (28–216)	Heel: 0 Middle foot: 1 Foremost part: 4	Partial necrosis: 0 Wound dehiscence: 1	CLTI: 5
Non-TCC group (our study) *N* = 8	52.3 days (24–109)	149 (66–270)	Heel: 2 Middle foot: 3 Foremost part: 2	Partial necrosis: 3 Wound dehiscence: 1	CLTI: 5
Santanelli et al 6 *N* = 14	1 month: 7 2 months: 1(excluding fracture cases)	73.5 (28–192)	Heel: 11 Middle foot: 3	No data	Trauma: 7 Tumor: 7
Han et al 7 *N* = 26	6 weeks	133.2 (22–486)	Heel: 11 Middle foot: 6 Foremost part: 6 Whole plantar: 3	Partial necrosis and delayed healing: 4	Trauma: 25 Other: 1
Abdelfattah et al 8 *N* = 18	4 weeks (excluding fracture cases)	115.5 (28–240)	Heel: 11 Middle foot: 5 Foremost part: 2	Partial necrosis: 1 Wound dehiscence: 1	CLTI: 4 Trauma: 10 Tumor: 3 Scar: 1
Lee et al 9 *N* = 33	No data	No data	Heel: 14 Middle foot: 13 Foremost part: 5 Ankle: 6	Partial necrosis: 4 Total necrosis: 3 Wound dehiscence: 6	CLTI: 33

Abbreviations: CLTI, chronic limb-threatening ischemia; TCC, total contact cast.

## Discussion

Patients with CLTI often have many complications and are unable to walk for an extended time because of plantar scars, even before surgery. Therefore, extended bed rest following surgery can lead to a decline in muscle strength, which may affect walking ability. Thus, walking should be resumed as soon as possible. In this study, the group that used TCC postoperatively had a shorter weighted onset period than the group that did not. These findings suggest that TCC is effective in preventing muscle weakness in patients who have undergone free flap foot surgery. Several reports of cases in which the sole was reconstructed using a free flap have noted that full weight-bearing occurred 1 month after the surgery ( Table 5 ). 6 7 8 9

A concern regarding the use of TCC in patients with CLTI is the increased complications of pressure ulcers and infection. 16 In-line with this, an erosion of approximately 1 cm was observed in one patient; however, it resolved conservatively, and there were no major complications.

Tickner et al recommended not using a TCC unless the systolic ankle pressure was ≥80 mm Hg. 13 In the current study, we used the SPP test and found that SPP and ankle pressure were similar. However, SPP fluctuates during dialysis, and the value may not be reliable in hemodialysis patients. 17 Although some of our patients had an SPP of 29 mm Hg, no complications occurred. Therefore, sponges to prevent pressure ulcers may be effective. In addition, replacing the TCC and checking the wound after 1 week was effective in preventing major complications.

A limitation of this study was the small number of patients, which may explain why no significant differences were observed. However, there were almost no TCC-related complications in the TCC group, and the results of this study showed that when using a TCC, the wound area was protected, and no problems arose when the full weight was applied 2 weeks after free flap reconstruction surgery. The inclusion of more patients in the study is unlikely to alter the present finding of fewer complications in the TCC group. Even without TCCs, complications may not increase if weight-bearing is started early, approximately 2 weeks after surgery. In this study, we could not deny this possibility. Another limitation of this study is that Asians tend to have smaller body frames and less load pressure compared with Westerners; thus, these results may not be generalizable to patients with large weight-bearing. We also believe that it is important to analyze each part of the foot in foot reconstruction, but the number of cases in this study was small and it was not possible to separate the results by part. We consider this study to be an exploratory study, and additional research is necessary in the future.

Patients with CLTI have a longer non-loading period, which increases patient stress. This problem can be alleviated by using a TCC postoperatively. Postoperative use of a TCC after free flap foot reconstruction may lead to an earlier hospital discharge and allow for early weight-bearing of the affected limb after surgery. In the future, randomized studies comparing the TCC and non-TCC groups should be conducted.

### Conclusion

TCCs were indicated in cases where plantar ulcers were reconstructed using free flaps and were useful in shortening the non-loading period without increasing the risk of postoperative complications. Further research is needed to confirm this conclusion.
